# Transcriptomic and miRNA–Target Pathway Analysis of the DLK1-DIO3 Imprinted microRNA Cluster in Chronic Lymphocytic Leukemia

**DOI:** 10.3390/diseases14090320

**Published:** 2026-09-03

**Authors:** Georgios S. Markopoulos, Yannis V. Simos, Konstantinos I. Tsamis, Lampros Lakkas, Eleftheria Hatzimichael, Eleni Kapsali, Dimitrios Peschos, Leonidas Benetatos

**Affiliations:** 1 Department of Physiology, Faculty of Medicine, School of Health Sciences, University of Ioannina, 45110 Ioannina, Greece; gmarkop@uoi.gr (G.S.M.); isimos@uoi.gr (Y.V.S.); ktsamis@uoi.gr (K.I.T.); l.lakkas@uoi.gr (L.L.); dpeschos@uoi.gr (D.P.); 2 Department of Hematology, Faculty of Medicine, School of Health Sciences, University of Ioannina, 45110 Ioannina, Greece; ehatzim@uoi.gr (E.H.); ekapsali@uoi.gr (E.K.)

**Keywords:** chronic lymphocytic leukemia, DLK1-DIO3 locus, microRNAs, B-cell receptor signaling, transcriptomics

## Abstract

Background/Objectives: Chronic lymphocytic leukemia (CLL) is a heterogeneous B-cell malignancy in which B-cell receptor signaling, microenvironmental interactions, genomic lesions, and epigenetic deregulation cooperate to shape disease behavior. The imprinted DLK1-DIO3 locus at chromosome 14q32 contains the largest human miRNA cluster and has been implicated in cancer-related regulatory networks; however, its contribution to CLL remains incompletely defined. Methods: In the present study, we investigated the potential involvement of DLK1-DIO3 miRNAs in CLL biology by integrating public transcriptomic datasets with miRNA-centered pathway analysis. GSE70830 was used as a discovery dataset and GSE66117 as a supportive validation cohort to identify genes consistently downregulated in CLL compared with normal B cells. Results: The analyses identified 1236 and 2145 downregulated genes, respectively, and their intersection yielded a 345-gene consensus set. The overlap was significantly greater than expected by chance (odds ratio 2.33; *p* = 1.55 × 10^−31^). This set was used as gene-filter input for DIANA-miRPath v3.0 analysis of the DLK1-DIO3 miRNA cluster, identifying eight KEGG pathways mainly involving B-cell receptor/NF-κB signaling, cell adhesion, leukocyte transendothelial migration, and glycan-related processes. DIANA-miRPath v4.0 provided pathway-centered refinement. GSE216258 miRNA analysis did not show generalized locus-wide upregulation, while a GSE12366 sensitivity analysis showed that 32 of the 345 genes overlapped the strongest naïve-memory B-cell differentiation signatures. The absence of uniform locus-wide upregulation suggests that these data do not establish a direct link between DLK1-DIO3 activation and generalized repression of the 345-gene set. Future studies are needed to identify gene- and pathway-specific effects. Conclusions: Overall, our findings support an association between DLK1-DIO3 miRNA target/pathway annotations and CLL-relevant transcriptional programs, providing a hypothesis-generating framework that warrants further experimental validation.

## 1. Introduction

Chronic lymphocytic leukemia (CLL) is a heterogeneous lymphoid malignancy characterized by the proliferation and accumulation of mature CD5+ B-cells [1]. CLL is pathogenetically distinguished by the presence or absence of mutations in the immunoglobulin heavy chain variable region (IGHV) genes, indicating memory B-cell or pre-germinal center origin, respectively. However, CLL pathogenesis is more complex, involving microenvironment interaction with B-cells, structural genomic aberrations, and epigenetic changes. Moreover, a vast number of driver somatic mutations accumulate in malignant cells, providing genetic heterogeneity; unmutated CLL carries more driver mutations than mutated CLL. Driver mutations cluster across several different pathways, providing interpatient heterogeneity. These pathways include NOTCH1 signaling, BCR and TLR signaling, the MAPK-ERK pathway, NF-kappaB signaling, chromatin modifiers, cell cycle, metabolism, inflammation, MYC and Wnt signaling, DNA damage response, and RNA splicing [2,3].

MicroRNAs (miRNAs) are short non-coding RNAs, ~22 nt long, with the potential to promote translation repression and mRNA destabilization through binding to complementary sequences in the 3′ untranslated region (3′ UTR) of target mRNAs, thereby localizing to target gene promoters and enhancers to regulate transcription, RNA processing and chromatin accessibility. A functional characteristic of miRNAs is that a single miRNA is able to target several hundred different target genes, whereas a single target mRNA can be targeted by different miRNAs [4,5]. More than half of the miRNAs are located in introns (and occasionally exons) of protein-coding genes and are transcribed, under the action of RNA polymerase II, with their host genes. The remaining are intergenic and transcribed independently of their host genes. Their transcription is controlled by transcription factors (TFs) and epigenetic modifications. Approximately 10% of miRNAs are broadly expressed among most tissues, whereas another ~10% are cell-type specific and under the control of tissue-specific TFs [5,6,7]. Genomic regions containing miRNA genes are often deleted, amplified, or translocated in human cancers, thus affecting their expression and the consequent downstream target mRNA expression. MiRNAs are also able to form complex networks with known potent oncogenes, such as MYC, enhancing each other’s expression and promoting oncogenesis [8]. Epigenetic modifications affecting the 3D chromatin conformation, a common feature in oncogenesis, modulate miRNA expression. For example, CpG islands of promoters of tumor-suppressive miRNAs tend to be hypermethylated in cancer, leading to their aberrant silencing. Moreover, miRNA interaction with EZH2 or MLL histone methyltransferases forms feedback pathways promoting altered histone methylation levels, such as H3K27me3 and H3K4me3, respectively, and oncogenesis [9,10]. Further, dysregulation of miRNA biogenesis machinery leads to aberrant expression of nascent miRNAs with defective activity. Finally, miRNA activity can be modulated by competing endogenous RNAs or by mutations of miRNA-binding sites at the 3′UTR of the target mRNA, with the final result being malignant transformation [4,5]. The involvement of miRNAs in CLL pathogenesis was first reported more than two decades ago when Calin and colleagues reported that mir-15 and mir-16 are located at chromosome 13q14, a region frequently deleted in CLL, and are also deleted or downregulated in the majority of CLL cases [11]. Later studies based on genome-wide expression analyses also provided evidence that miRNA expression is associated with established prognostic factors, such as IGHV mutations, and is able to identify patients with shorter time to first treatment, suggesting an overall prognostic impact of miRNAs in CLL biology, whereas they can be used as therapeutic targets in the CLL context [12,13].

DLK1-DIO3 represents one of the largest imprinted clusters in mammals, located at chromosome 14q32 in humans and 12qF in mice, a region conserved between the two species. That particular imprinted domain contains paternally expressed protein-coding genes (DLK1, RTL1, DIO3, and BEGAIN) and maternally expressed protein-coding genes (MEG3 (GTL2), MEG8 (RIAN), and antisense RTL1). The imprinting status of the locus is regulated by the germline-derived intergenic differentially methylated region (IG-DMR) required for the parental-specific expression of the cluster and acts as a bipartite element with two antagonistic cis-regulatory elements within the IG-DMR, ensuring the germline-specific DNA methylation pattern at DLK1-DIO3 [14,15]. Importantly, the DLK1-DIO3 locus contains 54 miRNAs, thus representing the largest one in the human genome. Aberrant expression of these miRNAs is implicated in the pathogenesis of several benign diseases, as well as solid and blood cancers. Their effect is achieved through modulation of important signaling pathways, cytokine signaling cascades, and epigenetic modifications, and it has been proposed that they can be used as biomarkers for the diagnosis and prognosis of several cancers [16,17,18].

The aim of our study was to investigate the putative functional role and overall involvement of DLK1-DIO3 miRNAs in CLL using an integrative transcriptomic and pathway-based approach. By integrating two public transcriptomic datasets, we derived a 345-gene consensus downregulated CLL set and used this gene set to interrogate the functional landscape of the DLK1-DIO3 miRNA cluster. Gene-restricted DIANA-miRPath v3.0 analysis highlighted CLL-relevant pathways centered on B-cell receptor (BCR)/NF-κB signaling and adhesion/migration-related processes, whereas pathway-centered v4.0 analysis was used for further refinement. Additional analyses of GSE216258 and GSE12366 were used to assess CLL-versus-normal miRNA expression and the potential contribution of normal B-cell differentiation, respectively.

## 2. Methods

### 2.1. Public Datasets and Study Design

We used four public GEO resources with complementary roles. GSE70830 included transcriptomic profiles from 10 CLL samples and 5 normal peripheral-blood CD19+ B-cell samples and served as the discovery mRNA dataset [19]. GSE66117 contained processed expression data from 47 CLL samples and 5 normal/control B-cell samples and served as an independent cross-dataset validation cohort [20]. The five GSE66117 controls consisted of one memory B-cell sample (GSM1614703), one naïve B-cell sample (GSM1614704), and three additional CD19+ B-cell controls (GSM1614705-GSM1614707). GSE216258 comprised 30 Affymetrix GeneChip miRNA 3.0 arrays (6 Asian CLL, 9 Asian normal, 9 Western CLL, and 6 Western normal) and was used for direct miRNA-expression analysis [21]. GSE12366 was used as an independent normal B-cell differentiation reference [22,23]. Complete sample annotations are provided in Supplementary Table S1.

To assess whether physiological B-cell differentiation could contribute to the 345-gene CLL-associated pattern, we performed a targeted sensitivity analysis using the direction-specific GSE12366 naïve-versus-memory B-cell signatures available in the C7 ImmuneSigDB collection [22,23]. The 345-gene set was intersected with the GSE12366_NAIVE_VS_MEMORY_BCELL_UP and GSE12366_NAIVE_VS_MEMORY_BCELL_DN signatures, and the direction of differentiation-associated expression was recorded for each overlapping gene (public datasets and samples used are presented in Supplementary Table S1).

### 2.2. Derivation of a Downregulated Gene CLL Signature

For the discovery step, we used the processed differential-expression file corresponding to the Normal-versus-CLL comparison from GSE70830. Genes were classified as downregulated in CLL when they showed a negative log2 fold change and q < 0.05.

For GSE66117, the deposited FPKM expression matrix was analyzed using 47 CLL samples and the 5 controls described above. Expression values were transformed as log2(FPKM + 1), and CLL and pooled controls were compared using a two-sided Welch *t*-test. *p*-values were adjusted across all 22,249 matrix rows using the Benjamini–Hochberg procedure. Genes with adjusted *p* < 0.05 and lower expression in CLL were retained, yielding 2145 downregulated genes. Because the control set contains only one memory and one naïve B-cell sample, subgroup-specific differential-expression testing against these individual controls was not considered statistically appropriate.

The two downregulated gene lists were harmonized at the gene-symbol level and intersected to define a cross-dataset consensus set. To quantify the robustness of this overlap, we defined a shared testable set of genes with finite effect estimates in both datasets and calculated the expected overlap under independence, Fisher/hypergeometric enrichment, Jaccard index, Pearson and Spearman effect-size correlations, and directional concordance. Complete gene-level outputs are provided in Supplementary Tables S2–S4 and S10.

### 2.3. Independent miRNA-Expression Assessment in GSE216258

Raw CEL files from GSE216258 were mapped to probesets using the Affymetrix miRNA 3.0 platform design. Probe intensities were processed using an RMA-style workflow comprising normal-exponential background correction, quantile normalization, log2 transformation, and median-polish probeset summarization [24]. The resulting fold changes were benchmarked against the deposited GSE216258 results before downstream interpretation. Because the array annotation is based on miRBase v17, historical probeset names were reconciled with current mature-miRNA nomenclature using MIMAT accessions.

**Figure 1:**
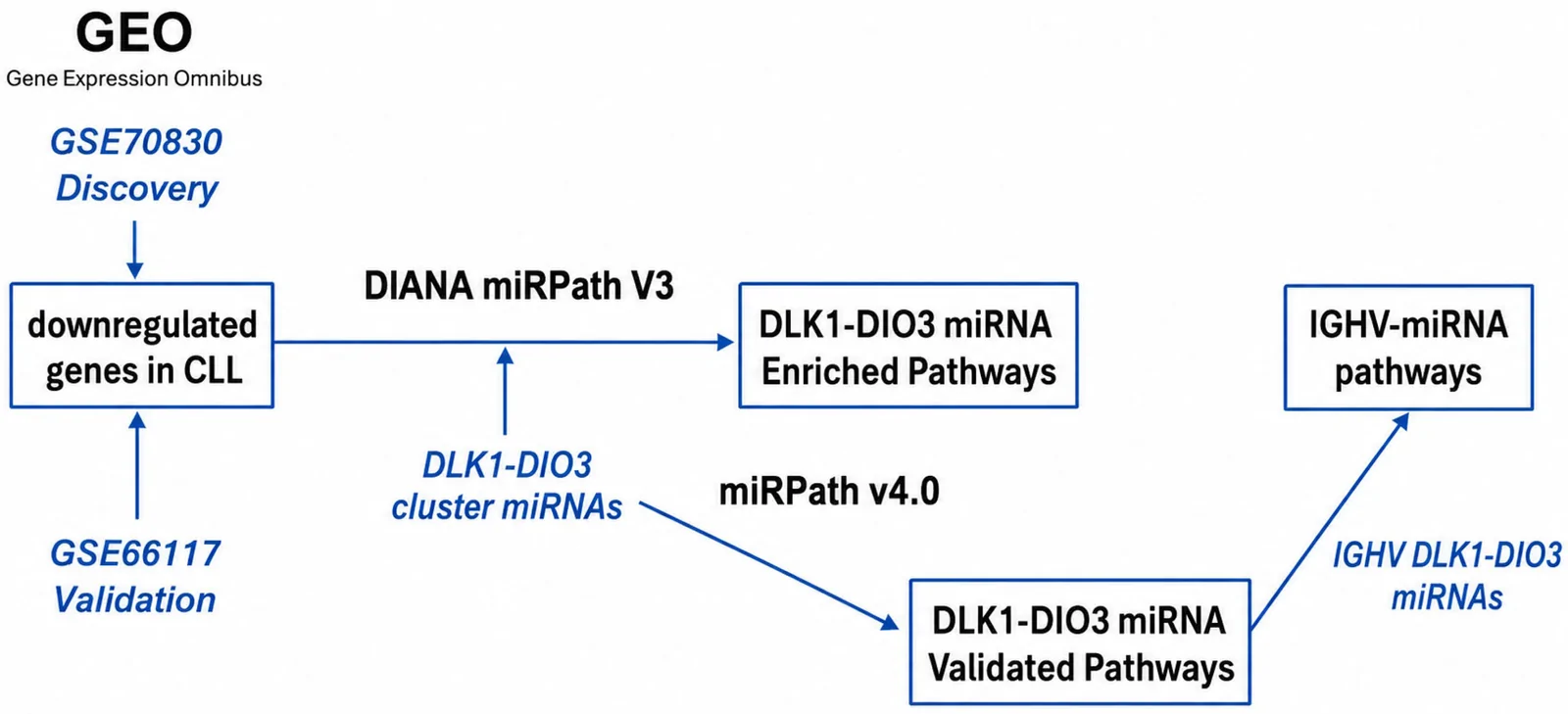
Analytical workflow for the identification of DLK1-DIO3 miRNA-associated pathways in CLL. Downregulated genes in CLL were identified using two public GEO datasets: GSE70830 as the discovery dataset and GSE66117 as the validation dataset. The resulting consensus downregulated gene signature was used as gene-filter input in DIANA-miRPath v3.0 together with the DLK1-DIO3 cluster miRNAs, leading to the identification of DLK1-DIO3 miRNA-enriched pathways. These pathways were further evaluated using DIANA-miRPath v4.0 as a refinement step, with additional annotation of pathways associated with the IGHV-related DLK1-DIO3 miRNA subset. The workflow summarizes the transition from transcriptomic repression in CLL to whole-cluster pathway enrichment and focused IGHV-miRNA pathway interpretation.

Two complementary miRNA analyses were performed: (i) the 7 IGHV-associated DLK1-DIO3 miRNAs highlighted by Bryant et al. (miR-543, miR-495-3p, miR-409-3p, miR-411-3p, miR-410-3p, miR-493-5p, and miR-493-3p) and (ii) all 69 mature-miRNA probesets mapping to the analyzed 14q32 interval [25]. Asian and Western cohorts were first analyzed separately using two-sided Welch tests. A population-adjusted disease effect was then estimated using expression-CLL status + population, together with a disease-by-population interaction model. Benjamini–Hochberg correction was applied separately across the seven-miRNA panel and the 69-locus probesets (Figure 1).

### 2.4. miRPath Analysis

To investigate the functional landscape potentially regulated by the DLK1-DIO3 miRNA cluster in CLL, pathway enrichment analysis was performed using DIANA-miRPath v3.0 [26]. The analysis included the full DLK1-DIO3 miRNA set, while the 345-gene consensus downregulated CLL set was used as the expressed-gene filter. This design restricted the pathway analysis to miRNA–target relationships occurring within the reproducibly downregulated transcriptomic compartment. The pathway-level *p*-values and mapped genes returned by the analysis are reported in Supplementary Table S5.

To refine the biologic architecture of the pathways identified in the primary analysis, a secondary pathway-centered analysis was performed using DIANA-miRPath v4.0 [27]. The broader pathway output was examined to further explore the contribution of individual DLK1-DIO3 miRNAs and their putative target relationships within relevant functional categories. To connect the pathway-centered analysis with IGHV-associated biology, we annotated the v4.0 output for the seven IGHV-associated DLK1-DIO3 miRNAs described by Bryant et al. [25] (Supplementary Tables S6 and S7).

## 3. Results

### 3.1. Identification of Downregulated Transcripts in CLL

We first interrogated GSE70830 as the discovery dataset to define genes transcriptionally repressed in CLL relative to normal peripheral-blood CD19+ B cells. Application of the predefined filtering criteria identified 1236 genes that were significantly downregulated in CLL (Supplementary Table S2). The complete output contained finite effect estimates and *p*/q-values within the expected numerical range.

To examine whether this pattern could be reproduced in an unrelated cohort, we analyzed GSE66117 as an independent validation dataset. Analysis of the processed expression matrix identified 2145 genes with significantly lower expression in CLL than in the pooled control samples (Supplementary Table S3). Because the control group contains one memory B-cell sample, one naïve B-cell sample, and three additional CD19+ controls, this dataset was used for supportive cross-dataset validation rather than as an age- and differentiation-matched case-control cohort.

**Figure 2:**
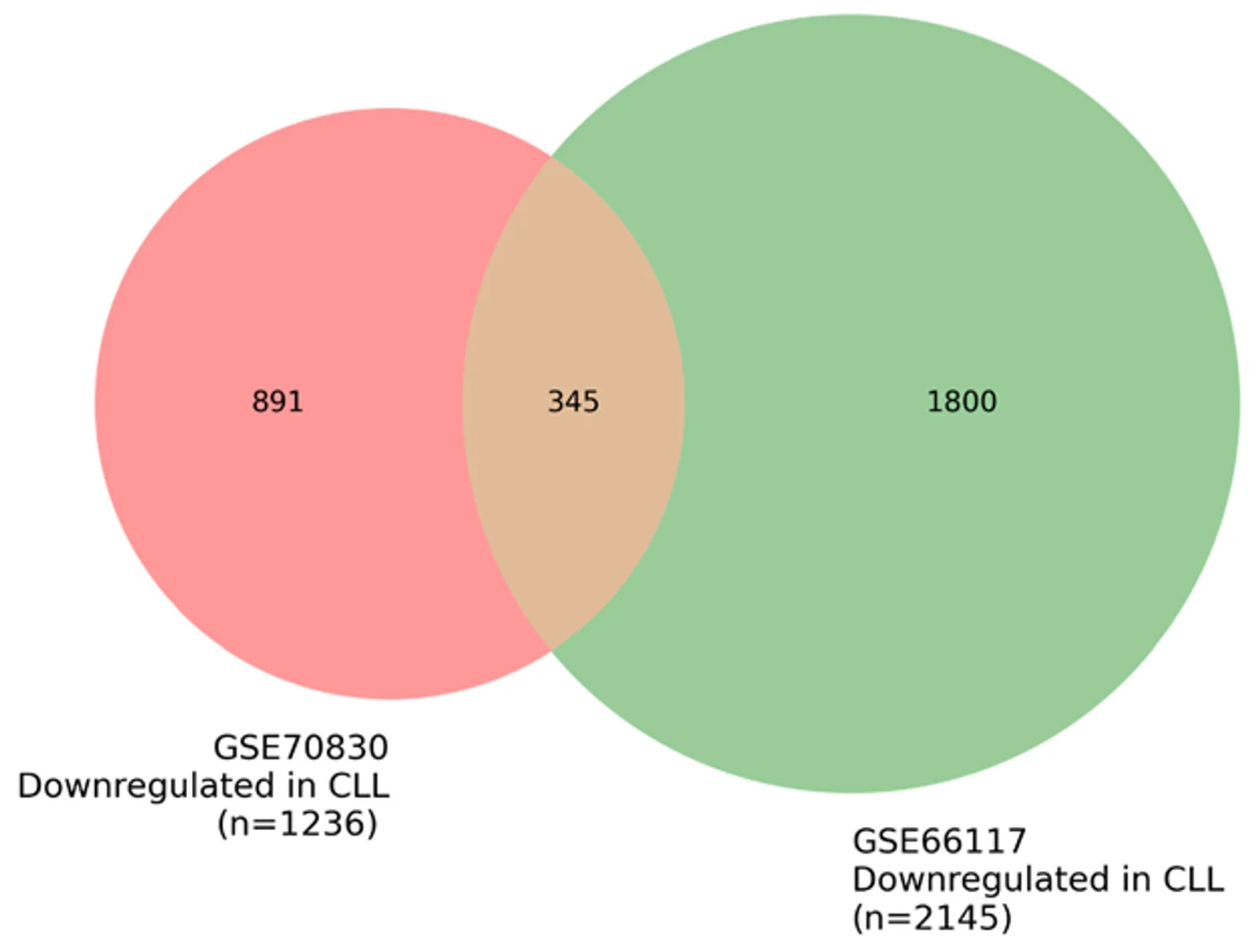
Venn diagram of downregulated genes identified in GSE70830 and GSE66117.

Intersection of the two downregulated gene sets yielded 345 genes consistently downregulated in CLL across both datasets (Figure 2; Supplementary Table S4). Within the shared testable universe of 12,600 genes, 1181 GSE70830 genes and 2063 GSE66117 genes met the downregulation criteria, with 345 shared genes. The observed overlap was 1.78-fold greater than expected under independence (Fisher odds ratio = 2.33; one-sided *p* = 1.548 × 10^−31^; Jaccard index = 0.119). Across all shared genes, effect-size concordance was statistically significant but modest (Pearson r = 0.136, *p* = 3.065 × 10^−53^; Spearman ρ = 0.032, *p* = 2.796 × 10^−4^), while 803/1181 (68.0%) of GSE70830-downregulated genes testable in GSE66117 showed the same negative direction (Supplementary Figure S1; Supplementary Table S10).

The biological plausibility of the consensus set was supported by the presence of several genes with recognized relevance to B-cell identity, signaling, adhesion, and leukemic cell biology, including EBF1, CR1, ITGA4, TRIB2, SMAD3, MYC, NT5E, and PLD4. Reduced EBF1 expression is compatible with altered B-cell identity, whereas ITGA4/CD49d and NT5E/CD73 are linked to microenvironmental retention, immune regulation, and clinical behavior in CLL [28,29,30]. The recurrence of these genes across two independent transcriptomic resources supports the reproducibility of the 345-gene CLL-associated downregulated set.

The 345 genes were therefore defined as a cross-dataset consensus downregulated set, reflecting the statistically significant overlap between independent cohorts while acknowledging the modest genome-wide effect-size concordance.

The GSE12366 differentiation sensitivity analysis identified 32 of the 345 genes (9.3%) within the strongest naïve-memory B-cell signatures (Supplementary Table S11). A total of 18 were represented in the naïve-higher signature and 14 in the memory-higher signature, whereas 313 genes (90.7%) were not represented in either top signature. Thus, normal B-cell differentiation can contribute to a measurable subset of the 345-gene pattern, but the cross-dataset signal is not explained solely by the biggest naïve-memory differences in this independent reference.

### 3.2. Independent Assessment of DLK1-DIO3 miRNA Expression in CLL

Analysis of the 30 raw GSE216258 miRNA arrays first reproduced the characteristic miR-4485 CLL-versus-normal signal reported in the deposited dataset (Asian CLL/normal 10.12-fold; Western CLL/normal 2.34-fold) and closely reproduced the subgroup fold changes of DLK1-DIO3 probes represented in the processed GEO output, supporting the validity of probe mapping and normalization.

All seven IGHV-associated DLK1-DIO3 miRNAs were represented on the array after mapping historical miRBase v17 names by MIMAT accession. None reached significance after Benjamini–Hochberg correction in the Asian, Western, or population-adjusted analyses (Supplementary Table S8; Supplementary Figure S2). miR-409-3p showed a nominal decrease in Asian CLL (fold change = 0.486; *p* = 0.0366), but this did not remain significant after correction across the seven-miRNA panel.

Across the 69 mature-miRNA probesets mapping to the analyzed 14q32 interval, the median population-adjusted fold change was 0.971, with 29 positive and 40 negative effects. Only miR-299-5p remained significant after correction across the 69 probesets (population-adjusted fold change = 0.785; *p* = 5.92 × 10^−4^; FDR = 0.0408), and its expression was decreased in CLL (Supplementary Table S9). These data indicate that generalized CLL-associated upregulation of the DLK1-DIO3/14q32 miRNA locus is not a uniform feature of this independent cohort.

### 3.3. DLK1-DIO3 miRNA Pathway Analysis Identifies CLL-Relevant Signaling and Microenvironment-Associated Programs

**Figure 3:**
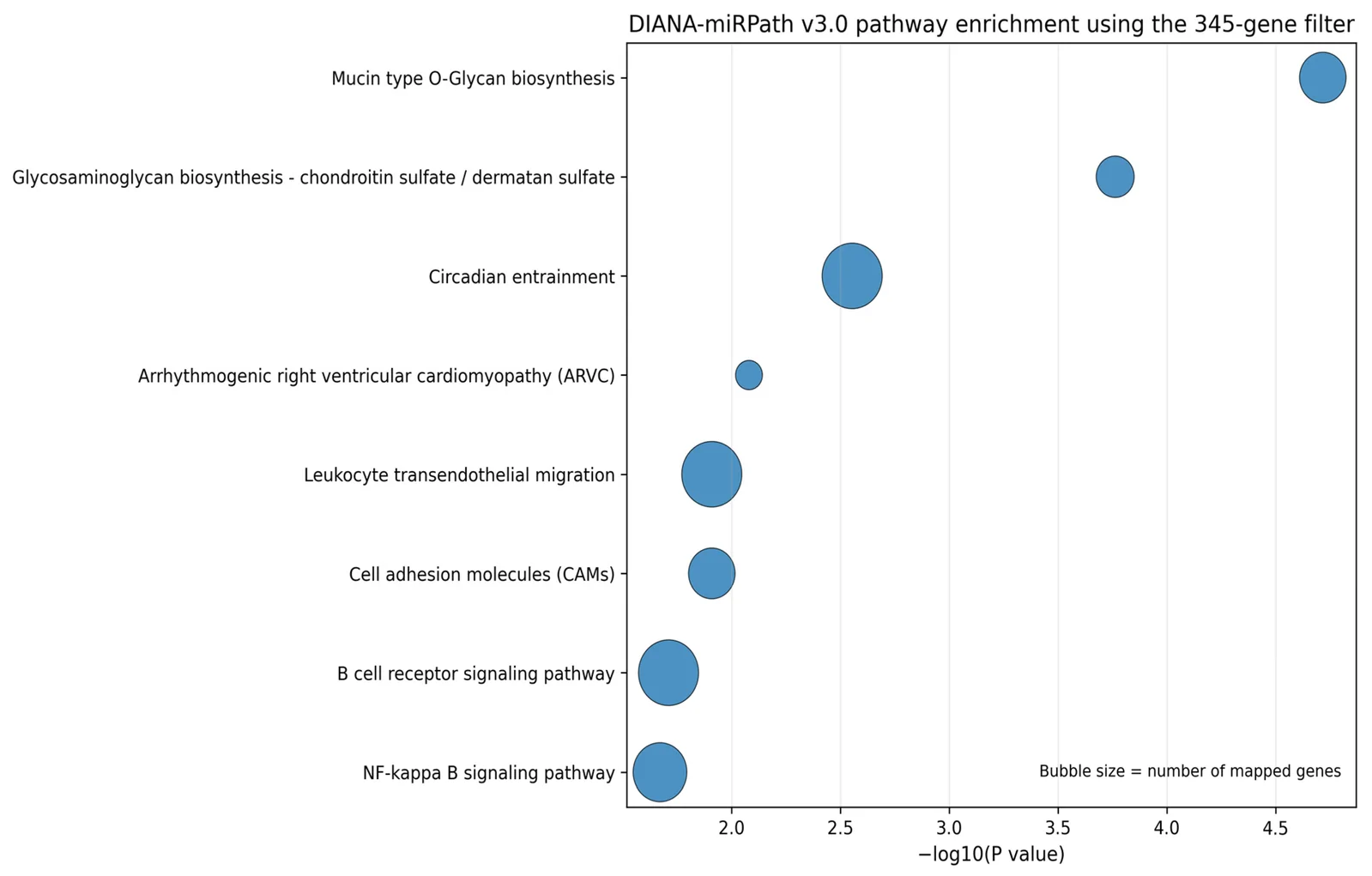
KEGG pathways identified by DIANA-miRPath v3.0 using the 345-gene consensus downregulated CLL set as the expressed-gene filter. The x-axis represents pathway enrichment as −log10(*p*-value), whereas bubble size corresponds to the number of mapped genes contributing to each pathway.

Using DIANA-miRPath v3.0 with the 345-gene consensus downregulated CLL set as the expressed-gene filter, eight KEGG pathways were identified with pathway-level *p* < 0.05 (Figure 3; Supplementary Table S5). These pathways converged on two main biological themes relevant to CLL pathobiology: core leukemic signaling and microenvironment-related cellular interaction. The identified pathways included B-cell receptor signaling, NF-κB signaling, cell adhesion molecules, leukocyte transendothelial migration, mucin-type O-glycan biosynthesis, glycosaminoglycan biosynthesis, circadian entrainment, and arrhythmogenic right ventricular cardiomyopathy (ARVC).

Among the most biologically relevant pathways were B-cell receptor signaling and NF-κB signaling, indicating a potential link between DLK1-DIO3 miRNA target annotations and signaling programs central to CLL cell activation and survival. The mapped genes included CR2, BLNK, DAPP1, MALT1, VAV3, TRAF5, and BIRC3. In parallel, cell adhesion molecules and leukocyte transendothelial migration included JAM3, ITGB2, ESAM, CYBB, and VAV3, highlighting pathways involved in cellular adhesion, trafficking, and interaction with the tissue microenvironment [31,32,33].

Inspection of the genes contributing to the pathways further supported their biological relevance to CLL. The B-cell receptor signaling pathway included CR2, BLNK, DAPP1, MALT1, and VAV3; the NF-κB pathway included TRAF5, BLNK, MALT1, and BIRC3; cell adhesion molecules included JAM3, ITGB2, and ESAM; and leukocyte transendothelial migration included JAM3, CYBB, VAV3, ITGB2, and ESAM. These mappings identify candidate miRNA–target relationships within pathways central to CLL biology.

The enrichment of mucin-type O-glycan and glycosaminoglycan biosynthesis pathways suggests that the transcriptional footprint associated with DLK1-DIO3 miRNA targets may also extend to cell-surface glycosylation and extracellular interaction programs. Circadian entrainment was also identified. ARVC was retained in the pathway output but was driven by a single mapped gene (JUP) and was therefore interpreted cautiously rather than as evidence of disease-specific cardiac biology.

Taken together, the v3.0 analysis identifies a pathway-level pattern centered on BCR/NF-κB signaling, adhesion/migration, and glycan-related processes within the reproducibly downregulated CLL gene set. These results provide a functional framework for prioritizing specific DLK1-DIO3 miRNA–target relationships for experimental testing.

The pathways identified by miRPath v3.0 were subsequently examined using DIANA-miRPath v4.0 in a pathway-centered manner (Supplementary Table S6). This secondary analysis was used as a refinement strategy to further resolve the contribution of individual cluster miRNAs and their putative targets within broader functional categories.

The v4.0 output included broad cancer- and signaling-related categories such as focal adhesion, proteoglycans in cancer, PI3K-Akt signaling, MAPK signaling, and pathways in cancer. These results provide additional pathway context for the DLK1-DIO3 miRNA cluster, but the 345-gene-filtered v3.0 analysis remains the primary pathway analysis of the study.

Focused annotation of the v4.0 output showed that five members of the Bryant-derived seven-miRNA panel (miR-495-3p, miR-409-3p, miR-411-3p, miR-410-3p, and miR-493-5p) were represented across multiple pathway categories (Supplementary Table S7). This links previously reported IGHV-associated 14q32 miRNAs with the pathway themes identified in the present analysis, while the GSE216258 results provide the direct CLL-versus-normal assessment of miRNA expression.

## 4. Discussion

MiRNA dysregulation in CLL may affect key targets such as BCL2, CCND1, TP53, MYC, and PTEN and modulate signaling pathways including B-cell receptor signaling, PI3K/AKT, JAK/STAT, Wnt, NF-κB, and NOTCH, thereby influencing cell proliferation, apoptosis, and treatment response [34,35].

Besides their putative involvement in CLL pathogenesis, miRNAs may have predictive value for treatment response. Among miRNAs associated with complete remission and undetectable minimal residual disease after immunochemotherapy, several members of the DLK1-DIO3 locus have been reported, including miR-412, miR-134, and miR-494 [36].

MiRNAs also have prognostic value in CLL and have been associated with overall survival, time to first treatment, and progression-free survival. Several DLK1-DIO3 miRNAs, including miR-412, miR-323-3p, miR-665, miR-376b-3p, and miR-370, have been linked to clinical outcome measures [37,38].

The present transcriptomic analysis identified a statistically non-random 345-gene overlap across two independent CLL datasets (odds ratio 2.33; *p* = 1.548 × 10^−31^), supporting a reproducible downregulated transcriptional component in CLL. The additional GSE12366 sensitivity analysis indicates that normal B-cell differentiation contributes to part of this signal, but only 32 of the 345 genes were represented among the strongest curated naïve-memory signatures. Thus, the replicated set provides a robust starting point for pathway interrogation while retaining the expected heterogeneity of cross-cohort CLL-versus-control comparisons.

Our pathway results implicate BCR/NF-κB signaling and adhesion/migration programs, which are central to CLL pathogenesis and microenvironmental dependence [31,32,33,39]. These findings are also consistent with Bryant et al., who showed that DLK1-DIO3 miRNAs are strongly associated with IGHV-related transcriptional differences and experimentally demonstrated regulation of the GAB1 3′UTR by miR-409-3p and miR-411-3p [25]. The present study extends this framework by identifying pathway-level relationships between DLK1-DIO3 target annotations and a reproducibly downregulated CLL gene set.

The absence of generalized locus-wide upregulation in the GSE216258 dataset is an important constraint on causal interpretation. Activation of the DLK1-DIO3 cluster miRNAs may not be the main regulatory mechanism associated with the 345-gene set downregulation. It remains possible that a proportion of these genes are regulated by mechanisms unrelated to DLK1-DIO3 miRNAs. At the same time, this cohort-level result does not exclude context-dependent activity of individual 14q32 miRNAs in molecularly defined CLL subsets, particularly given the IGHV-associated differences reported by Bryant et al. [25]. Accordingly, the pathway analysis should be interpreted as prioritizing plausible miRNA–target pathway relationships rather than demonstrating a locus-wide causal repression program. DNA methylation may represent a plausible alternative or cooperating mechanism. The DLK1-DIO3 domain is controlled by imprinting-associated differentially methylated regions, including the IG-DMR, so changes in methylation may influence transcriptional activity across the locus [14,15]. Conversely, methylation changes affecting promoters or other regulatory regions of genes within the 345-gene set could contribute directly to their reduced expression, independently of miRNA activity. Coordinated hypo- and hypermethylation events have been described in leukemia [20]. The present study does not integrate matched DNA-methylation, miRNA, and mRNA measurements from the same CLL samples, and therefore cannot determine the relative contribution of locus methylation, target-gene methylation, and miRNA-mediated regulation. We suggest that a matched multi-omic design in future studies will be required to resolve these alternatives.

Glycosylation is known to be associated with CLL, accounting for the low levels of B-cell receptor surface expression, especially the unmutated subtype, enhancing signal transduction via increased tyrosine phosphorylation and mediating the crosstalk between cancer cells and the microenvironment [40,41,42]. Aberrant glycosylated forms on the surface of cancer cells make them attractive therapeutic targets for drug delivery systems [43]. Thus, our findings place glycosylation and microenvironment-related processes among the pathway contexts associated with DLK1-DIO3 miRNA target annotations. Given the absence of uniform locus-wide upregulation, these associations should not be interpreted as evidence that the pathways are under direct DLK1-DIO3 miRNA control. Defective glycosylation of the mi chain and CD79a has been shown to contribute to reduced surface BCR expression in CLL, while more recent work demonstrated that sialylation regulates CLL migration through post-translational modification of CD49d, coupling glycan remodeling to microenvironmental trafficking [41,42]. Because endothelial and stromal interactions are major determinants of CLL survival and drug resistance, the coexistence in our analysis of glycan pathways with CAM/transendothelial migration pathways provides evidence for a possible coordinated, rather than incidental, microenvironmental signature [31].

Mucin-type O-glycosylation has a role in protein stability regulation and is essential for proper development, differentiation, and growth of cells. Mucin-type O-glycosylation exhibits substrate function for non-enzymatic sugar-binding proteins, inducing signal transduction affecting cell growth and apoptosis, cell-to-cell interactions, and cell–matrix interactions as well. Abnormal mucin-type O-glycosylation has been associated with oncogenesis, whereas mucin-type O-glycosylation therapeutic targeting has been explored through various strategies [44,45]. Our findings suggest that cell-surface glycosylation and extracellular interaction programs are relevant contexts for selected DLK1-DIO3 miRNA–target relationships. We believe that these observations warrant future functional testing of individual miRNA-gene interactions rather than therapeutic inference at the level of the entire locus.

Circadian rhythm maintains cellular homeostasis operating through transcription-translation feedback loops involving the essential clock genes, including CLOCK, BMAL1, NPAS2, PERs, CRYs, RORs, REV-ERBs, DECs, CK1ε, NONO and TIM, with multiple components represented in miRNA target annotations. It has been shown that perturbation of circadian rhythm is associated with a higher risk of hematological malignancies occurring by affecting the major cancer hallmarks. In particular, aberrant expression of BMAL1 and PER1-2 genes disrupts MYC and CCND1 expression, enhancing cell proliferation and inhibiting apoptosis [46,47]. We would nevertheless interpret the circadian signal cautiously. Rather than claiming a direct circadian mechanism for DLK1-DIO3 miRNAs in CLL, the present data suggest that transcriptional programs intersecting calcium signaling, kinase activity, and MYC-linked cellular timing may constitute a pathway context associated with the locus [46].

ARVC emerged in the pathway analysis but was driven by a single mapped gene (JUP). Although arrhythmogenic complications are clinically relevant in hematologic malignancies and in CLL patients receiving BTK inhibitors [48,49], the present pathway result is insufficient to support a direct cardiac mechanism and is therefore not considered a core biological conclusion of this study.

DLK1-DIO3 miRNAs have also been implicated in resistance to BTK inhibition through regulation of the PTEN/AKT/mTOR pathway. In particular, several 14q32 miRNAs have been reported to increase in resistant B-cell lymphoid models and to contribute to PTEN downregulation [50]. Together with the molecular heterogeneity of targeted-therapy resistance in CLL [51], these findings provide additional rationale for studying specific DLK1-DIO3 miRNA–target relationships in signaling and microenvironmental contexts.

The interaction between miRNAs and their target genes forms complex regulatory networks in which individual miRNAs may influence multiple targets and several co-expressed miRNAs can converge on the same pathway [6]. This network concept is particularly relevant to the DLK1-DIO3 locus. Bryant et al. reported that 14q32 miRNAs represent a substantial component of the differentially expressed miRNA repertoire between IGHV-mutated and unmutated CLL and are linked to BCR, Wnt, and Ras-related transcriptional programs [25]. Our v4.0 annotation further showed that five members of the Bryant-derived panel are represented within several pathway categories identified here.

Several limitations of the present study should be acknowledged. The mRNA datasets differ in cohort composition, control source, processing, and available clinical annotation, and GSE66117 does not contain sufficient numbers of matched naïve and memory controls for subgroup-specific differential-expression testing. The GSE12366 sensitivity analysis partly addresses this issue but cannot substitute for an age- and differentiation-matched CLL cohort. Reference resources such as GenomicScape further illustrate the extent of transcriptional remodeling across normal B-cell differentiation [52]. In addition, the miRNA and mRNA measurements were obtained from different cohorts, so inverse within-sample miRNA–target correlations could not be assessed. The miRPath v3.0 pathway analysis uses the 345-gene expressed-gene filter, whereas v4.0 was used as a broader pathway-centered refinement step. As with all database-driven miRNA–target analyses, the identified relationships require experimental confirmation in primary CLL samples or appropriate functional models.

From a translational perspective, miRNA-directed therapeutic strategies remain an active area of development [53,54]. The present findings support prioritization of specific DLK1-DIO3 miRNA–target pairs rather than therapeutic targeting of the locus as a whole. Matched miRNA/mRNA profiling, experimentally validated binding evidence, and functional perturbation will be important next steps for identifying the most biologically and clinically relevant interactions.

## 5. Conclusions

Our findings support an association between the DLK1-DIO3 miRNA locus and CLL-relevant transcriptional programs. Integration of two independent transcriptomic datasets identified a reproducible 345-gene downregulated set, and gene-filtered pathway analysis highlighted BCR/NF-κB signaling, adhesion/migration, and glycan-related processes. Independent CLL-versus-normal miRNA profiling did not indicate uniform upregulation of the 14q32 locus, while normal B-cell differentiation accounted for a subset of the replicated gene pattern. Importantly, the absence of uniform 14q32 upregulation limits causal interpretation: the present data do not demonstrate that generalized DLK1-DIO3 activation drives repression of the 345-gene set, and alternative mechanisms, including DNA methylation, remain possible. Together, these data provide a focused hypothesis-generating framework for mechanistic validation of specific DLK1-DIO3 miRNA–target relationships in CLL.

## Data Availability

The public source datasets are available from NCBI GEO under accession numbers GSE70830, GSE66117, GSE216258, and GSE12366. The generated data from source datasets are available in Supplementary Materials.

## Supplementary Materials

The following supporting information can be downloaded at: https://www.mdpi.com/article/10.3390/diseases14090320/s1, Figure S1: Cross-dataset effect-size concordance between GSE70830 and GSE66117, Figure S2: Independent CLL-versus-normal expression assessment of the seven IGHV-associated DLK1-DIO3/14q32 miRNAs in GSE216258; Table S1: Public datasets, analytical roles, and sample annotations, Table S2: GSE70830 genes significantly downregulated in CLL, Table S3: GSE66117 genes significantly downregulated in CLL, Table S4: Replicated 345-gene cross-dataset downregu-lated set, Table S5: DIANA-miRPath v3.0 pathway results using the 345-gene expressed-gene filter, Table S6: DIANA-miRPath v4.0 pathway-centered output, Table S7: Annotation of the Bryant IGHV-associated seven-miRNA panel within the DIANA-miRPath v4.0 output, Table S8: Inde-pendent GSE216258 raw-array assessment of the seven IGHV-associated DLK1-DIO3 miRNAs, Table S9: GSE216258 assessment of 69 mature-miRNA probesets mapping to the DLK1-DIO3/14q32 interval, Table S10: Cross-dataset concordance, validation checks, and provenance, Table S11: Normal B-cell differentiation sensitivity analysis using GSE12366-derived naïve-memory signatures.

## References

[1] Hallek M. (2025). Chronic Lymphocytic Leukemia: 2025 Update on the Epidemiology, Pathogenesis, Diagnosis, and Therapy. Am. J. Hematol..

[2] Knisbacher B.A., Lin Z., Hahn C.K., Nadeu F., Duran-Ferrer M., Stevenson K.E., Tausch E., Delgado J., Barbera-Mourelle A., Taylor-Weiner A. (2022). Molecular map of chronic lymphocytic leukemia and its impact on outcome. Nat. Genet..

[3] Bosch F., Dalla-Favera R. (2019). Chronic lymphocytic leukaemia: From genetics to treatment. Nat. Rev. Clin. Oncol..

[4] Lin S., Gregory R.I. (2015). MicroRNA biogenesis pathways in cancer. Nat. Rev. Cancer.

[5] Ding S., Wang P. (2025). The Life of MicroRNAs: Biogenesis, Function and Decay in Cancer. Biomolecules.

[6] Bracken C.P., Scott H.S., Goodall G.J. (2016). A network-biology perspective of microRNA function and dysfunction in cancer. Nat. Rev. Genet..

[7] Kim H., Lee Y.Y., Kim V.N. (2025). The biogenesis and regulation of animal microRNAs. Nat. Rev. Mol. Cell Biol..

[8] Benetatos L., Vartholomatos G., Hatzimichael E. (2014). Polycomb group proteins and MYC: The cancer connection. Cell. Mol. Life Sci..

[9] Benetatos L., Voulgaris E., Vartholomatos G., Hatzimichael E. (2013). Non-coding RNAs and EZH2 interactions in cancer: Long and short tales from the transcriptome. Int. J. Cancer.

[10] Benetatos L., Vartholomatos G. (2013). MicroRNAs mark in the MLL-rearranged leukemia. Ann. Hematol..

[11] Calin G.A., Dumitru C.D., Shimizu M., Bichi R., Zupo S., Noch E., Aldler H., Rattan S., Keating M., Rai K. (2002). Frequent deletions and down-regulation of micro-RNA genes miR15 and miR16 at 13q14 in chronic lymphocytic leukemia. Proc. Natl. Acad. Sci. USA.

[12] Calin G.A., Ferracin M., Cimmino A., Di Leva G., Shimizu M., Wojcik S.E., Iorio M.V., Visone R., Sever N.I., Fabbri M. (2005). A MicroRNA signature associated with prognosis and progression in chronic lymphocytic leukemia. N. Engl. J. Med..

[13] Ali A., Mahla S.B., Reza V., Hossein A., Bahareh K., Mohammad H., Fatemeh S., Mostafa A.B., Leili R. (2024). MicroRNAs: Potential prognostic and theranostic biomarkers in chronic lymphocytic leukemia. EJHaem.

[14] Weinberg-Shukron A., Youngson N.A., Ferguson-Smith A.C., Edwards C.A. (2023). Epigenetic control and genomic imprinting dynamics of the Dlk1-Dio3 domain. Front. Cell Dev. Biol..

[15] Aronson B.E., Scourzic L., Shah V., Swanzey E., Kloetgen A., Polyzos A., Sinha A., Azziz A., Caspi I., Li J. (2021). A bipartite element with allele-specific functions safeguards DNA methylation imprints at the Dlk1-Dio3 locus. Dev. Cell.

[16] Benetatos L., Voulgaris E., Vartholomatos G. (2012). DLK1-MEG3 imprinted domain microRNAs in cancer biology. Crit. Rev. Eukaryot. Gene Expr..

[17] Benetatos L., Hatzimichael E., Londin E., Vartholomatos G., Loher P., Rigoutsos I., Briasoulis E. (2013). The microRNAs within the DLK1-DIO3 genomic region: Involvement in disease pathogenesis. Cell. Mol. Life Sci..

[18] Manodoro F., Marzec J., Chaplin T., Miraki-Moud F., Moravcsik E., Jovanovic J.V., Wang J., Iqbal S., Taussig D., Grimwade D. (2014). Loss of imprinting at the 14q32 domain is associated with microRNA overexpression in acute promyelocytic leukemia. Blood.

[19] Liao W., Jordaan G., Nham P., Phan R.T., Pelegrini M., Sharma S. (2015). Gene expression and splicing alterations analyzed by high throughput RNA sequencing of chronic lymphocytic leukemia specimens. BMC Cancer.

[20] Kushwaha G., Dozmorov M., Wren J.D., Qiu J., Shi H., Xu D. (2016). Hypomethylation coordinates antagonistically with hypermethylation in cancer development: A case study of leukemia. Hum. Genom..

[21] Liu P., Wang K., Li J., Ogasawara M.A., Xia Z., Wierda W.G., Keating M.J., Li Y., Huang P. (2024). Global miRNA profiling reveals key molecules that contribute to different chronic lymphocytic leukemia incidences in Asian and Western populations. Haematologica.

[22] Longo N.S., Lugar P.L., Yavuz S., Zhang W., Krijger P.H.L., Russ D.E., Jima D.D., Dave S.S., Grammer A.C., Lipsky P.E. (2009). Analysis of somatic hypermutation in X-linked hyper-IgM syndrome shows specific deficiencies in mutational targeting. Blood.

[23] Godec J., Tan Y., Liberzon A., Tamayo P., Bhattacharya S., Butte A.J., Mesirov J.P., Haining W.N. (2016). Compendium of Immune Signatures Identifies Conserved and Species-Specific Biology in Response to Inflammation. Immunity.

[24] Carvalho B.S., Irizarry R.A. (2010). A framework for oligonucleotide microarray preprocessing. Bioinformatics.

[25] Bryant D., Smith L., Rogers-Broadway K.R., Karydis L., Woo J., Blunt M.D., Forconi F., Stevenson F.K., Goodnow C., Russell A. (2023). Network analysis reveals a major role for 14q32 cluster miRNAs in determining transcriptional differences between IGHV-mutated and unmutated CLL. Leukemia.

[26] Vlachos I.S., Zagganas K., Paraskevopoulou M.D., Georgakilas G., Karagkouni D., Vergoulis T., Dalamagas T., Hatzigeorgiou A.G. (2015). DIANA-miRPath v3.0: Deciphering microRNA function with experimental support. Nucleic Acids Res..

[27] Tastsoglou S., Skoufos G., Miliotis M., Karagkouni D., Koutsoukos I., Karavangeli A., Kardaras F., Hatzigeorgiou A.G. (2023). DIANA-miRPath v4.0: Expanding target-based miRNA functional analysis in cell-type and tissue contexts. Nucleic Acids Res..

[28] Li L., Zhang D., Cao X. (2024). EBF1, PAX5, and MYC: Regulation on B cell development and association with hematologic neoplasms. Front. Immunol..

[29] Pozzo F., Tissino E., Zucchetto A., Gattei V. (2024). CD49d in chronic lymphocytic leukemia: A molecule with multiple regulation layers. Comment to “Sialylation regulates migration in chronic lymphocytic leukemia”. Haematologica.

[30] Allard D., Chrobak P., Bareche Y., Allard B., Tessier P., Bergeron M.A., Johnson N.A., Stagg J. (2022). CD73 Promotes Chronic Lymphocytic Leukemia. Cancers.

[31] Cerreto M., Foà R., Natoni A. (2023). The Role of the Microenvironment and Cell Adhesion Molecules in Chronic Lymphocytic Leukemia. Cancers.

[32] Meijers R.W.J., Muggen A.F., Leon L.G., de Bie M., van Dongen J.J.M., Hendriks R.W., Langerak A.W. (2020). Responsiveness of chronic lymphocytic leukemia cells to B-cell receptor stimulation is associated with low expression of regulatory molecules of the nuclear factor-κB pathway. Haematologica.

[33] Diop F., Moia R., Favini C., Spaccarotella E., De Paoli E., Bruscaggin L., Spina A., Terzi-di-Bergamo V., Arruga L., Tarantelli F. (2020). Biological and clinical implications of BIRC3 mutations in chronic lymphocytic leukemia. Haematologica.

[34] Chu A., Soto F., Hurtado R., Tirado C.A. (2026). Epigenetics in B-CLL. Int. J. Genom..

[35] Doghish A.S., Abulsoud A.I., Elshaer S.S., Abdelmaksoud N.M., Zaki M.B., El-Mahdy H.A., Ismail A., Fathi D., Elsakka E.G. (2023). miRNAs as cornerstones in chronic lymphocytic leukemia pathogenesis and therapeutic resistance—An emphasis on the interaction of signaling pathways. Pathol.-Res. Pract..

[36] Duroux-Richard I., Gagez A.-L., Alaterre E., Letestu R., Khalifa O., Jorgensen C., Leprêtre S., Tchernonog E., Moreaux J., Cartron G. (2022). MiRNA profile at diagnosis predicts treatment outcome in patients with B-chronic lymphocytic leukemia: A FILO study. Front. Immunol..

[37] Aghayan A.H., Arab A., Haddadi S., Mirazimi Y., Hosseinzadeh A., Mohtashami T., Atashi A. (2025). Investigating the prognostic value of non-coding RNAs in chronic lymphocytic leukemia: Insights from a systematic review and meta-analysis. BMC Cancer.

[38] Nano E., Reggiani F., Amaro A.A., Monti P., Colombo M., Bertola N., Ferrero F., Fais F., Bruzzese A., Martino E.A. (2024). MicroRNA Profiling as a Predictive Indicator for Time to First Treatment in Chronic Lymphocytic Leukemia: Insights from the O-CLL1 Prospective Study. Non-Coding RNA.

[39] El-Daly S.M., Bayraktar R., Anfossi S., Calin G.A. (2020). The Interplay between MicroRNAs and the Components of the Tumor Microenvironment in B-Cell Malignancies. Int. J. Mol. Sci..

[40] Krysov S., Potter K.N., Mockridge C.I., Coelho V., Wheatley I., Packham G., Stevenson F.K. (2010). Surface IgM of CLL cells displays unusual glycans indicative of engagement of antigen in vivo. Blood.

[41] Natoni A., Cerreto M., De Propris M.S., Del Giudice I., Soscia R., Peragine N., Intoppa S., Milani M.L., Guarini A., Foà R. (2023). Sialylation regulates migration in chronic lymphocytic leukemia. Haematologica.

[42] Vuillier F., Dumas G., Magnac C., Prevost M.-C., Lalanne A.I., Oppezzo P., Melanitou E., Dighiero G., Payelle-Brogard B. (2005). Lower levels of surface B-cell-receptor expression in chronic lymphocytic leukemia are associated with glycosylation and folding defects of the μ and CD79a chains. Blood.

[43] Diniz F., Coelho P., Duarte H.O., Sarmento B., Reis C.A., Gomes J. (2022). Glycans as Targets for Drug Delivery in Cancer. Cancers.

[44] Zhang Y., Sun L., Lei C., Li W., Han J., Zhang J., Zhang Y. (2022). A Sweet Warning: Mucin-Type O-Glycans in Cancer. Cells.

[45] Sun L., Zhang Y., Li W., Zhang J., Zhang Y. (2023). Mucin Glycans: A Target for Cancer Therapy. Molecules.

[46] Motiei M., Abu-Dawud R., Relógio A., Assaf C. (2024). Circadian rhythms in haematological malignancies: Therapeutic potential and personalised interventions. eBioMedicine.

[47] Sanford A.B.A., da Cunha L.S., Machado C.B., de Pinho Pessoa F.M.C., dos Santos Silva A.N., Ribeiro R.M., Moreira F.C., de Moraes Filho M.O., de Moraes M.E.A., de Souza L.E.B. (2022). Circadian Rhythm Dysregulation and Leukemia Development: The Role of Clock Genes as Promising Biomarkers. Int. J. Mol. Sci..

[48] Al Mubaid A., Baba M., Vinod V., Siddiqui O., Marsh-Kates K., Hussein A. (2026). Chemotherapy-Induced Arrhythmogenic Right Ventricular Cardiomyopathy. JACC Case Rep..

[49] Tomasulo E., Itsara A., Haigney M., Rosing D.R., Ahn I.E., Peer C., Kozel B.A., Luperchio T., Ge G., Figg W.D. (2026). Sudden death and asymptomatic arrhythmia in chronic lymphocytic leukemia patients treated with ibrutinib. Heart Rhythm.

[50] Kapoor I., Bodo J., Hill B.T., Almasan A. (2021). Cooperative miRNA-dependent PTEN regulation drives resistance to BTK inhibition in B-cell lymphoid malignancies. Cell Death Dis..

[51] Blombery P., Chatzikonstantinou T., Gerousi M., Rosenquist R., Gaidano G., Pospisilova S., Roberts A.W., Birkinshaw R.W., Rossi D., Scarfo L. (2025). Resistance to targeted therapies in chronic lymphocytic leukemia: Current status and perspectives for clinical and diagnostic practice. Leukemia.

[52] Kassambara A., Rème T., Jourdan M., Fest T., Hose D., Tarte K., Klein B. (2015). GenomicScape: An Easy-to-Use Web Tool for Gene Expression Data Analysis. Application to Investigate the Molecular Events in the Differentiation of B Cells into Plasma Cells. PLoS Comput. Biol..

[53] Wang Z., Peng Y., Zhou H., Zhang M., Ju D., Chen Z. (2025). MiRNA-based drugs: Challenges and delivery strategies. Appl. Microbiol. Biotechnol..

[54] Di Martino M.T., Tagliaferri P., Tassone P. (2025). MicroRNA in cancer therapy: Breakthroughs and challenges in early clinical applications. J. Exp. Clin. Cancer Res..

