# Supplementary material for: Transcriptomic and miRNA–Target Pathway Analysis of the DLK1-DIO3 Imprinted microRNA Cluster in Chronic Lymphocytic Leukemia
Source: Diseases. 2026 Sep 3;14(9):320. doi: 10.3390/diseases14090320 (PMC13605451; doi:10.3390/diseases14090320)
Supplement: Supplementary file 1 [file diseases-14-00320-s001.zip › Supplementary_Figure_Legends.pdf]

Supplementary Figure Legends

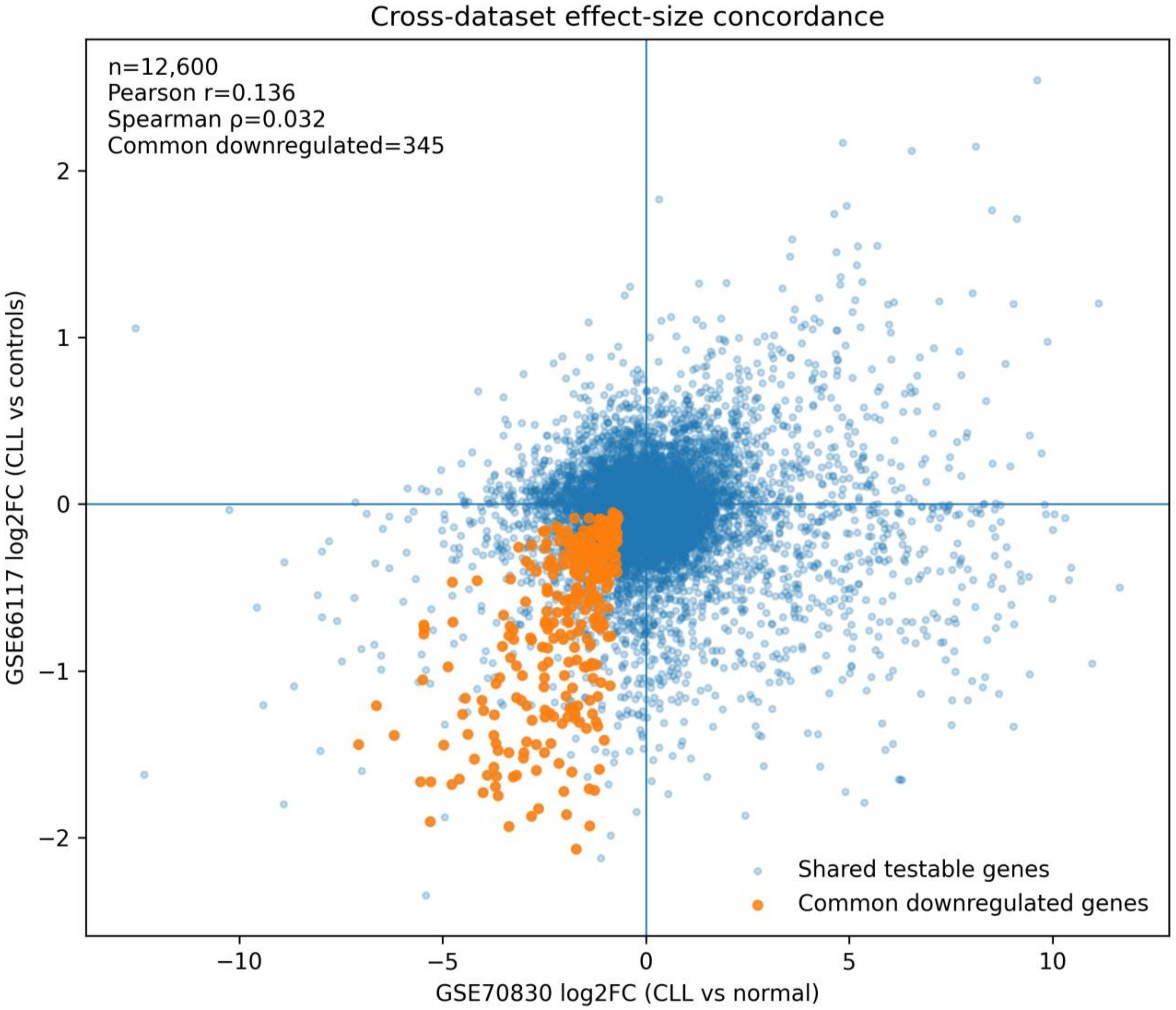

Supplementary Figure S1. Cross-dataset effect-size concordance between GSE70830 and GSE66117. Each point represents one of the 12,600 unique gene symbols testable in both datasets, with axes showing log2 fold change for CLL versus normal/control B cells in GSE70830 and GSE66117, respectively. Genes belonging to the 345-gene cross-dataset consensus downregulated set are highlighted. Across all shared genes, Pearson correlation was r=0.136 (P=3.065×10^-53) and Spearman correlation was ρ=0.032 (P=2.796×10^-4), indicating statistically significant but quantitatively modest genome-wide effect-size concordance.

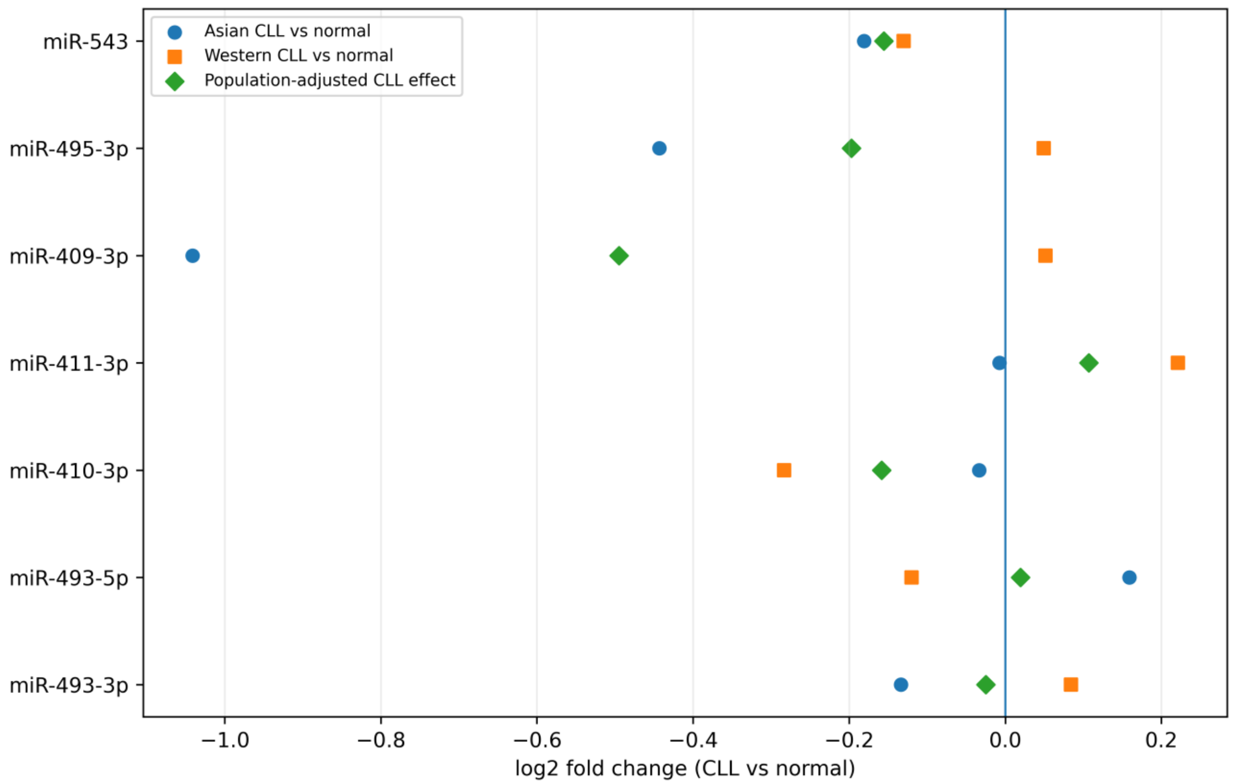

Supplementary Figure S2. Independent CLL-versus-normal expression assessment of the seven IGHV-associated DLK1-DIO3/14q32 miRNAs in GSE216258. The plot shows log2 fold changes for Asian CLL versus Asian normal B cells, Western CLL versus Western normal B cells, and the population-adjusted disease effect. The vertical reference line indicates no change (log2 fold change=0). Historical miRBase v17 array nomenclature was reconciled using mature-miRNA accessions. No member of the seven-miRNA panel was significant after Benjamini-Hochberg correction, and the panel did not show consistent CLL-associated upregulation across the two population strata.
